# You Understand, So I Understand: How a “Community of Knowledge” Shapes Trust and Credibility in Expert Testimony Evidence

**DOI:** 10.3390/bs15081071

**Published:** 2025-08-06

**Authors:** Ashley C. T. Jones, Morgan R. Haga

**Affiliations:** School of Psychology, The University of Southern Mississippi, College Drive #5025, Hattiesburg, MS 39406, USA; morgan.haga@usm.edu

**Keywords:** expert witness, forensic evaluation, witness credibility, community of knowledge effect, jury decision-making

## Abstract

Sloman and Rabb found support for the existence of the community of knowledge (CK) effect, which occurs when individuals are more likely to report understanding and being able to explain even fake scientific information when told that an expert understands the information. To date, no studies have been conducted that attempted to replicate original findings, let alone test the presence of the CK effect in realistic, legal scenarios. Therefore, Study One replicated original CK effect studies in a jury-eligible M-Turk sample (*N* = 291) using both Sloman and Rabb’s experimental stimuli as well as new stimuli. Study Two then tested the presence of the CK effect using scientific testimony in a mock court hearing from a forensic evaluator (*N* = 396). Not only did the CK effect improve laypeople’s perceptions of the scientific information in court, but it also improved their perceptions of the expert witness’s credibility, increased the weight assigned to the scientific information, and increased the weight assigned to the expert testimony. This effect was mediated by participants’ perceived similarity to the expert, supporting the theory behind the CK effect. These studies have important implications for the use of scientific information in court, which are discussed.

## 1. Community of Knowledge

In 2016, Sloman and Rabb proposed the existence of the “community of knowledge” effect, or CK effect. The effect occurs when an observer is told that another person understands how something works, resulting in the observer believing they also understand how it works without actually receiving an explanation. This newly identified effect, in isolation, is a simple social-cognitive psychology theory that explains how people rely on one another for information. However, when put into the legal context, the CK effect is an example of how insufficiently supported scientific testimony can influence decision-making in high-stakes legal cases. In the present study, we aimed not only to replicate the original CK effect study but test the influence of the CK effect in a fictitious legal case, including perceptions of trust and credibility, as well as on the decisions made that rely upon scientific testimony from an expert witness.

Sloman and Rabb (2016) tested the influence of others’ perceived understanding of novel scientific information in a series of four within-group studies utilizing Amazon Mechanical Turk samples. Participants read news briefs describing fictitious scientific findings (such as the existence of triangular lightning, glowing rocks, and humming stalactites), then measured perceived understanding and perceived receipt of an explanation for how the phenomenon worked. The news briefs varied in four ways across studies: the type of scientific finding that was discovered, the level of difficulty in understanding how the phenomenon worked, whether discovering scientists understood and could explain how it worked, and how accessible the scientific information was. Key findings demonstrated that participants were more likely to perceive themselves as understanding how the fictitious scientific finding worked when they were told that discovering scientists understood and could explain the scientific phenomenon. This phenomenon, coined the “community of knowledge” effect, describes the perception of understanding novel scientific information to such a high degree that individuals feel they can explain how the science works—without having received enough information to reasonably do so. The roots of the CK effect are found in the philosophical idea of a “community of knowledge”, which posits that ideas gain meaning within communities through several factors: the essence of the idea (Medin & Ortony, 1989), one’s own personal knowledge of the idea (Wilson, 2002), and the trustworthiness and believability of community members within which ideas are shared (Welbourne, 1981).

In these flagship studies, the CK effect was not influenced by the level of difficulty in understanding how the phenomenon worked. It was, however, moderated by the perceived accessibility of the scientific information—participants did not perceive themselves as able to understand how the novel scientific finding worked when they were told such information was inaccessible (Sloman & Rabb, 2016). This finding suggests the CK effect is not impervious to correction, which has important implications for how we understand the effect to operate. The ability to mitigate the CK effect by making clear the receiver does not have access to the scientific information is a quality shared with the “illusion of explanatory depth”, in which participants initially rate themselves as understanding something until they are asked to explain how it works, at which point they often realize they cannot—and subsequently reduce their ratings of perceived understanding (Rozenblit & Keil, 2002).

Beyond the four original studies by Sloman and Rabb (2016), virtually no other studies exist to help replicate and further define the CK effect. One study extended the CK effect to mitigation behaviors related to the COVID-19 pandemic and found that scientists’ reported understanding of the virus accounted for more variation in mitigation behaviors than an individual’s own understanding of the virus (Rabb et al., 2024). However, little else is known about the instances in which the CK effect is present or absent, nor the circumstances under which it strengthens or weakens, because of the absence of research in this area. Indeed, research has yet to empirically test the theoretical construct of the CK effect and whether perceptions of understanding are truly grounded in a perception of “community”. Thus, there is much left to be understood about the effect and how it influences various types of decision-making.

## 2. Source Credibility and the CK Effect

Source credibility has long been understood to influence perceptions of information. A critical review by Pornpitakpan (2006) summarizes expansive research on source credibility, concluding that sources with high credibility are more persuasive than those with low credibility. The CK effect, from a theoretical perspective, assumes that receivers of scientific information perceive themselves to either be within the same social circle as the source of scientific information (Sloman & Rabb, 2016) or perceive the source of information to be a social “thought leader” or authority figure they are affiliated with who has specialized knowledge (Rabb et al., 2024). This aspect of the CK effect and what influence source credibility has on the CK effect has yet to be empirically tested; such information may have implications for how the CK effect is anticipated and mitigated, particularly in decision-making contexts.

## 3. Scientific Information in the Courtroom

In criminal court trials, expert witnesses are often used to provide specialized knowledge that will help the jury and judge determine case facts and make decisions (Federal Rules of Evidence, 2000). Precedent was set in the landmark case *Daubert v. Merrell Dow Pharmaceuticals, Inc.* (1993) that judges should act as gatekeepers in court, only allowing experts to testify on scientific information grounded in reliable and valid methods, accepted by the scientific community, subjected to a peer-review process, and with a rate of error within an acceptable range. Despite this standard being set, research suggests the Daubert criteria are not always effective in protecting jurors from flawed science on their own. Gatowski et al. (2001) found in a survey of judges that while many of them agreed with the assigned role of “gatekeeper” to protect courts from faulty scientific testimony, the vast majority did not understand key scientific concepts well enough to identify and call out faulty scientific testimony when it exists. Kovera and McAuliff (2000) also conducted a study to determine judges’ ability to make evidence admissibility decisions using legal standards, particularly when flawed science was present, and when the judge had some type of formal scientific training (e.g., taken a graduate-level science course, completed a Continuing Education course on scientific methods). Researchers found that only 17% of judges allowed scientifically valid testimony into trial; judges with formal scientific training had higher admissibility ratings for the scientifically valid testimony, while judges with no formal scientific training had higher admissibility ratings for the scientifically flawed testimony. Research also suggests experts who use jargon or immaterial scientific information to explain their scientific methods may be viewed as more credible (Fernandez-Duque et al., 2015; Hopkins et al., 2016). It is possible the CK effect may be responsible for faulty science making it “past the gatekeeper” without contest (Vickers, 2005), because experts report understanding and trusting the faulty science, attorneys are less likely to raise objections regarding the veracity of its scientific basis, and jurors are less likely to doubt its legitimacy.

Scientific information in the courtroom is not immune to the influence of source credibility. Perceptions of expert credibility have been known to influence verdict and sentencing decisions (Cooper & Neuhaus, 2000; Cramer et al., 2011). Both perceptions of the expert’s knowledge and trustworthiness impact expert credibility (Cramer et al., 2009), along with an expert’s visual presentation, including eye contact and poise (Cramer et al., 2011). Most pertinent to the CK effect, Parrott et al. (2015) found that mock jurors rated the expert witness as more likable—a component of perceived credibility—when the expert had low versus high content knowledge. This stands in contrast to literature on source credibility and trust, which suggests that more credentials and more knowledge elicit more trust; however, this may be in line with the CK effect in that experts with less knowledge may be perceived as within a layperson’s community. More research is needed to determine which of these cases is true for the CK effect.

## 4. Current Studies

To contribute to the scarce literature on the CK effect and understand its role in legal settings, two experimental studies were conducted. The first study sought to replicate the original CK effect by Sloman & Rabb and extend the findings to understand the extent of its influence on layperson perceptions; the second study then tested the CK effect in the context of a mock court hearing to assess its influence, if any, in a realistic legal setting that relies upon accurate perceptions of scientific information. These studies will not only bridge the gap between classic social psychology and applied research, but assist forensic evaluators and attorneys in using research on the CK effect to improve their own expert testimony, effectively challenge experts whose testimony is vague or who utilize questionable scientific information, and to help attorneys and judges better identify cases of insufficiently supported scientific testimony. It may even help judges make sense of scientific information in the context of the Daubert criteria, especially when attorneys do not effectively challenge scientific testimony.

### 4.1. Study One

#### 4.1.1. Study One Method

This study sought to replicate and expand the original CK effect study. We expected to find the following:

**H1.**  *Similarly to Sloman and Rabb (2016), scientists’ understanding of fake scientific information would be associated with increased participant understanding of the scientific information, as well as perceived ability to explain*.

**H2.**  *In addition to original study outcomes, we expected to find that scientists’ understanding of the fake scientific information would be associated with increased participant believability of the scientific information, as well as trust in the scientific information*.

**H3.**  *Source quality would interact with this relationship in that the CK effect would be lower for low-quality sources, consistent with literature on source credibility and trust.*

##### Study One Participants

Participants were collected using Amazon Mechanical Turk (M-Turk) crowdsourcing marketplace, using the add-on Turk Prime to reach a more representative sample (Litman et al., 2017). To be eligible to participate, participants had to be at least 18 years old, have no felony convictions, and be designated a “master worker” by M-Turk, increasing the likelihood of obtaining high-quality data. A total of N = 300 participants were included in the original sample. It was determined a priori to remove participants who completed the survey in under 90 s. Thus, nine participants were removed from the original sample, resulting in a final sample of N = 291 participants. On average, the final sample completed the survey in 247 s. Participants’ ages ranged from 19 to 74, with an average age of 34.71 (SD = 10.82). The sample was primarily identified as male (55.3%) and Caucasian/White (71.5%). A full breakdown of sample demographics is provided in Table 1.

##### Study One Design and Procedure

A total of eight different news briefs were created for this study, which varied in three ways: the scientist’s reported understanding of the novel scientific finding, the news brief’s publication source quality, and the type of scientific finding described. This resulted in a 2 (Understanding: no vs. yes) × 2 (Publishing source: local newspaper vs. academic journal) × 2 (Phenomenon: melting rocks vs. humming stalactites) partial mixed factors design. Participants were randomly assigned to one of four possible conditions, each containing two news briefs and associated survey items. The two news briefs in each condition were “opposites” of one another—if participants were assigned to view the news brief describing humming stalactites reported by a local newspaper that scientists could understand, their second news brief then described melting rocks reported by an academic journal that scientists could not understand. This allowed us to maximize data collection across all eight news briefs while preserving the integrity of the data.

In order to replicate the original findings, the type of scientific phenomenon was manipulated. Half of the news briefs contained an adapted version of the “humming stalactites” phenomenon used by Sloman and Rabb (2016), while the other half contained a new phenomenon created for this specific study, specifically the occurrence of “melting rocks”. The condition adapted from Sloman and Rabb (2016) stated the following “…[a newspaper] reported the discovery of a cave formation that scientists have thoroughly explained. The authors of the study, Danica and Frith, gave a description of the unusual formation: The otherwise ordinary stalactites generate a continuous humming sound without being touched”. The melting rocks condition included the same types of information and number of sentences but described “seemingly common desert stone [that] turns into a liquid state when it comes into contact with water”. Each of the news brief vignettes is openly available on Open Science Framework (OSF). We manipulated the type of phenomenon presented, not because we expected the phenomenon to change perceptions of understanding, but to ensure findings could be generalized to different types of fake scientific information. An analysis of variance with phenomenon type as the independent variable indicated that the melting rock condition resulted in lower perceptions of ability to explain, *p* = 0.03, perceived trustworthiness, *p* < 0.001, and belief that the phenomenon actually happened, *p* < 0.001, compared to the humming stalactite condition. Although the reason for this difference is unclear, it may simply be an artifact of the fake scientific information chosen.

The second manipulation, expert understanding, was adapted from Sloman and Rabb (2016) and varied in whether or not the scientists in each scenario understood how the phenomenon worked. Half of the scenarios stated scientists fully understood the phenomenon and could explain how it worked. The other scenarios stated that the scientists who discovered the phenomenon did not understand, and could not explain, how it worked. To ensure a successful replication, the language for this manipulation was directly adapted from the original studies and included one sentence about whether the scientists understood how the phenomenon worked and whether they had an explanation of the underlying process of the phenomenon.

The last manipulation in the study, publishing source quality, was original to the present study. It was included to measure the extent to which the CK effect would be influenced by the peripheral cue of source quality. In the low-quality source condition, the phenomenon was reported by a local newspaper, whereas in the high-quality source condition, the phenomenon was reported by an academic journal. This manipulation included one sentence at the beginning of the vignette describing where the study was reported, along with a brief summary of the phenomenon and whether scientists understood it: “A June 26, 2015, study in the local newspaper Billings Herald reported the discovery of a cave formation that scientists have thoroughly explained”.

To recruit participants, a brief information statement outlining the project topic and aims was presented on the M-Turk website. Interested participants were directed to an online survey created using Qualtrics online survey platform, where they reviewed the informed consent detailing voluntary participation and minimal anticipated risks as a result of their participation. Consenting participants then viewed the assigned news briefs one at a time. After viewing each news brief, participants answered a series of questions related to the news brief. The survey in total took approximately five minutes to complete, and participants were compensated $0.60 for their time.

##### Study One Measures

Items were derived primarily from the original study by Sloman and Rabb (2016) to measure dependent variables. On a scale from 1 (Not well at all) to 5 (Extremely well), participants were asked about their perceived understanding of the scientific information (i.e., “How well do I understand how this phenomenon works?”), perceived ability to explain how the scientific information worked (i.e., “How well could I explain how this phenomenon works?”), perceived believability of the scientific information (i.e., “How well do I believe that this phenomenon exists?”), and perceived trust in the source of the information (i.e., “How well do I trust the source that published the research?”). We additionally asked participants several other researcher-derived items, including if they believed they received an actual explanation of how the scientific information worked (i.e., “Did you receive an explanation on how the phenomenon worked?”), from 1 (No explanation) to 5 (A complete, thorough explanation). Ranging from 1 (Not at all important) to 5 (Extremely important), participants were also asked how important it is for scientists to “Discover new things”, “Find new facts”, “Explain how things work”, and “Understand why something happens”. Finally, participants were asked demographic questions regarding their age, gender, and other demographic characteristics.

#### 4.1.2. Study One Results

All dependent measures met general assumptions for analysis of variance (ANOVA) testing. To prepare for analysis, we collapsed data across the phenomenon variable (as it was included for replication purposes and not anticipated to change participant perceptions). Because each participant viewed two opposite conditions, responses to survey items on the second condition were reverse-coded to create change scores for all dependent variables. Prior to reverse-coding, higher scores on the dependent measures represented higher scores of participant understanding; after reverse-coding, higher scores represented a bigger difference in participant understanding between the first and second news briefs. Change scores were analyzed as opposed to raw scores in Study One primarily because of the repeated-measures design; analyzing change scores allowed the analysis to be collapsed across time points and to primarily test between-subjects factors, which were the primary dependent variables of interest. The time point was simply an artifact of the phenomenon variable, which was tested separately. Descriptive statistics for all dependent measures are included in Table 2.

##### Study One Expert Understanding

A series of within-subjects ANOVA tests was performed to measure the effect of expert understanding on participants’ perceptions of the scientific information. Results indicated there was a significantly larger difference in participant understanding from when the scientist understood the scientific information (*M* = 3.11, *SE* = 0.05) to when the scientist did not understand the scientific information (*M* = 2.70, *SE* = 0.05), *F*(1, 287) = 40.64, *p* < 0.001, η^2^ = 0.12. In other words, the original CK effect was replicated. Additionally, differences in participants’ perceived ability to explain how the scientific information worked were significantly larger when participants were told scientists understood and could explain how it worked (*M* = 3.09, *SE* = 0.05) compared to when they were told scientists did not understand and could not explain how it works (*M* = 2.72, *SE* = 0.05), *F*(1, 287) = 30.98, *p* < 0.001, η^2^ = 0.10. There was a significantly larger difference in ratings of participants’ belief that the scientific information they read about actually existed when scientists understood the scientific information (*M* = 3.13, *SE* = 0.06) compared to differences in perceptions when scientists did not understand (*M* = 2.73, *SE* = 0.05), *F*(1, 288) = 27.48, *p* < 0.001, η^2^ = 0.09. Lastly, there was a significantly larger difference in participant trustworthiness of the scientific information when scientists understood the scientific information (*M* = 3.20, *SE* = 0.05) compared to the difference when scientists did not understand (*M* = 2.80, *SE* = 0.05), *F*(1, 287) = 31.70, *p* < 0.001, η^2^ = 0.10.

To ensure that participants did not misinterpret the news brief as providing an explanation of how the scientific information worked, an ANOVA was conducted examining the influence of expert understanding on participants’ perceptions of having received an actual explanation. Results indicated a significant difference in perceptions of having received an explanation in that there was a smaller difference in scores when scientists did not understand the novel scientific finding (*M* = 2.54, *SE* = 0.06) compared to when scientists understood the novel scientific finding (*M* = 3.21, *SE* = 0.06), *F*(1, 288) = 66.04, *p* < 0.001, η^2^ = 0.19. If participants misinterpreted the scientific findings’ description as an explanation, then participants would have thought they received an actual explanation across conditions, resulting in no significant differences. Therefore, the observed results are likely not because of a misinterpretation of the news briefs but rather changes in perceptions because of the CK effect.

##### Study One Source Quality

A between-subjects ANOVA was performed to measure the effect of source quality on perceptions of the scientific information. Results indicated there were no effects of source quality on participant understanding (*p* = 0.56), perceived ability to explain (*p* = 0.78), perceived believability (*p* = 0.46), or perceived trustworthiness of the scientific information (*p* = 0.32). These findings suggest that source quality alone had little influence on perceptions of the scientific information.

##### Study One Expert Understanding vs. Source Quality

**Figure 1:**
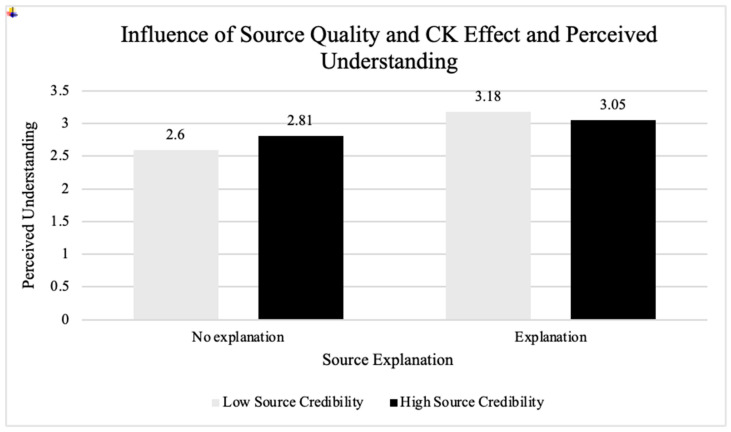
Influence of source quality and CK effect on perceived understanding.

A two-way between-subjects ANOVA test revealed an interaction between expert understanding and source quality on participant understanding, *F*(1, 287) = 6.85, *p* = 0.01, η^2^ = 0.02. Source quality appeared to moderate the effect of expert understanding on participant understanding; when the source quality was low, the CK effect was significant, with higher participant understanding when scientists understood the scientific information (*M* = 3.18, *SE* = 0.07) and lower participant understanding when scientists did not understand the scientific information (*M* = 2.60, *SE* = 0.06), *F*(1, 143) = 33.32, *p* < 0.001, η^2^ = 0.19. When the source quality was high, the CK effect was significant in the same direction, but weaker, with higher scores of participant understanding when scientists understood the scientific information (*M* = 3.05, *SE* = 0.06) and lower scores of participant understanding when scientists did not understand the scientific information (*M* = 2.81, *SE* = 0.06), *F*(1, 144) = 8.94, *p* < 0.01, η^2^ = 0.06 (see Figure 1). There was no interaction between source quality and expert understanding on perceived ability to explain (*p* = 0.16), perceived believability (*p* = 0.16), or perceived trustworthiness of the scientific information (*p* = 0.08).

#### 4.1.3. Study One Discussion

Study One is to our knowledge the first study to attempt replication of Sloman and Rabb (2016) and also extend results to other components central to the perception of scientific information, including source quality, believability, and trustworthiness. We found support for Hypothesis 1 in that participants’ understanding of and ability to explain the scientific information were higher when they were told experts understood and could explain the scientific information. We also extended our understanding of the influence of the CK effect; we found participants’ beliefs that the scientific information is real, as well as perceptions of the source’s trustworthiness, increased when experts reported understanding the scientific information, supporting Hypothesis 2. Lastly, we found evidence to support Hypothesis 3, although not in the predicted direction: results suggested an interaction between expert’s reported understanding of the novel scientific finding and the source quality in that the CK effect was stronger when the scientific information was reported by a local newspaper, compared to when it was reported by an academic journal.

The interaction between expert understanding and source quality suggests that the CK effect may be more prominent when there are fewer cues for information credibility or trustworthiness. A possible explanation is that source quality signals the “community” of people who understand the scientific information. Theoretical underpinnings of the CK effect suggest participants need to perceive a shared identity or community with the source of information to have higher perceptions of understanding (Sloman & Rabb, 2016). Similarly to Parrott et al. (2015), Study One found more favorable perceptions of the scientist when they were less credible--or the information came from a low-quality source. If participants perceived themselves as sharing the same community as the local newspaper, the CK effect may be stronger in this condition compared to the academic journal. We attempted to test this theory as part of Study Two.

An alternative explanation is that low-quality sources interact with expert understanding in a manner consistent with the Elaboration Likelihood Model (ELM) of persuasion (Petty & Cacioppo, 1986). The ELM suggests that when a person is highly interested in a topic, they are motivated to pay attention to factual information (called central cues) and actively learn more about the information. However, when a person is not motivated to make an informed decision, they rely on information that is not always pertinent to credibility or trustworthiness (peripheral cues) to make decisions about the information. In the present study, the CK effect was stronger when the source quality was lower. This finding would suggest that scientific information from a newspaper may act as a peripheral cue for source credibility, increasing the CK effect when present. A review by Hafdahl et al. (2021) outlined the ways in which the ELM guides juror decision-making specifically; thus, additional research on the CK effect and its relevance to the ELM is pertinent to understanding the effect from a theoretical perspective as well as how it applies to jury decision-making processes.

The primary limitation of Study One is that while it contributes to social psychological literature on the CK effect, it does not offer information on how the CK effect changes decision-making in real-world scenarios. Indeed, research to date on the CK effect has only focused on fake scientific phenomena, including weather events and rock formations in scenarios where the information has little importance. Research on the CK effect needs to be extended into areas examining high-stakes decision-making, such as legal settings, where scientific information is central to decision-making and can have dire consequences when performed ineffectively (Devine, 2012). We make an attempt to address this literature gap in Study Two.

Additional limitations include the fact that we did not account for impression management in the study design, preventing us from testing the extent to which impression management and a desire to appear “smart” contribute to the CK effect. Similarly, our study did not test the theoretical underpinnings of the CK effect with dependent variables that align with ELM (Petty & Cacioppo, 1986), preventing us from drawing conclusions about the social psychological theory driving the CK effect. We further did not test the influence of participant demographic variables on the presence of the CK effect. Another limitation is that while we included some manipulation checks, we did not include any measures of the strength of the source quality manipulation, nor did we include suspicion checks to determine if participants could tell the purpose of the research. Without these checks, it is difficult to determine if the lack of relationship between source quality and dependent variables is valid. Lastly, there is conceptual research on the CK effect surrounding its intersection with political values and aligned behavior (see Rabb et al., 2024), yet we did not hypothesize around or test the influence of political beliefs. These considerations must be rectified in future studies to ensure the CK effect is a true effect (not just a byproduct of impression management, political beliefs, or individual differences) and continue shaping our understanding of the conditions under which the CK effect exists.

### 4.2. Study Two

#### 4.2.1. Study Two Method

It is conceptualized that laypeople’s perceived understanding of scientific information when presented by an expert who reportedly understands the information is driven by a shared “community” within which information can be trusted (Sloman & Rabb, 2016), even if one may not understand it themselves. Therefore, to test the theory that the CK effect is stronger with low-quality sources because of increased perceptions of belonging to the same community, and to apply the CK effect to legal settings, we conducted Study Two. A scenario involving a mock court hearing with expert testimony by a forensic evaluator was chosen to assess the extent to which the replicated CK effect generalizes to expert testimony, which often relies upon complex scientific information and directly informs decision-making by jurors. We expected to find the following:

**H1.**  *The CK effect would be present in the context of expert testimony, in that participants’ understanding of, trustworthiness of, believability in, and ability to explain fictitious scientific information would be higher when the forensic evaluator reported understanding and being able to explain the scientific information.*

**H2.**  *There will be an interaction between expert understanding and expert credentials in that the CK effect will be stronger when the evaluator has less experience in the field compared to more because of hypothesized decreases in perceived social distance (similar to Study One).*

**H3.**  *Participant perceptions of social distance from the evaluator would be lower (therefore perceiving increased similarity to the evaluator), and perceptions of expert credibility would be lower, when the evaluator had less experience in their field.*

**H4.**  *The CK effect will be explained at least in part by perceived social distance from the evaluator, with lower perceived social distance (therefore perceiving increased similarity to the expert), leading to a stronger CK effect.*

##### Study Two Participants

A sample of jury-eligible participants was recruited using Prolific crowdsourcing platform. To be eligible for the study, participants were required to be 18 years or older, live in the United States, have no felony convictions, and speak English. A total of N = 400 participants consented to study participation. Four participants were excluded from the original sample because they did not complete all survey items, and all participants passed attention checks, resulting in a final sample of N = 396. There were approximately 32 participants who failed both manipulation checks; although we did not exclude participants for failing manipulation checks, this was taken into consideration in the discussion of study findings. Participants’ ages ranged from 18 to 77, with an average age of 37.62 (SD = 12.64). The sample was primarily identified as female (56.1%), White (69.9%), and non-Hispanic (88.9%). A full breakdown of sample demographics is provided in Table 3.

##### Study Two Design and Procedure

Eight new scenarios describing a mock court hearing in which a forensic evaluator testified about a defendant’s mental state at the time of the offense were created for this study. They varied in three ways: the evaluator’s reported understanding of a novel scientific technique used in the evaluation, the evaluator’s credentials, and the evaluator’s opinion as to whether the individual acted intentionally at the time of the incident. This resulted in a 2 (Understanding: no vs. yes) × 2 (Credentials: low vs. high) × 2 (Opinion: did not act intentionally vs. did act intentionally), between-subjects design. In contrast to Study One, participants were randomly assigned to one of eight conditions, each reading one vignette before answering survey questions; there was no repeated measures component to Study Two.

The vignette described a mock court hearing with expert testimony regarding a defendant’s mental state at the time of the alleged offense, with a forensic evaluator testifying about a mental state evaluation conducted with the defendant. In their testimony, the evaluator describes a fictitious novel scientific method of assessing defendants’ mental states (called Cognitive Intent Imaging) and their ultimate recommendation of the person’s mental state at the time of the alleged offense. While authors understand that forensic evaluators do not typically offer “ultimate opinions” regarding a defendant’s mental state as part of their evaluation, this information was included for research purposes to assess the extent to which participants agreed with the evaluator’s opinion. A final statement regarding the disposition of the case was also provided to participants. The first author, a trained forensic evaluator, reviewed the vignettes to maximize ecological validity while preserving experimental manipulations to answer the relevant research questions.

The first manipulation regarding the evaluator’s reported understanding of the novel scientific technique was used to test the influence of the CK effect on participants’ perceptions of scientific information in a legal context. This included one sentence describing whether the evaluator understood the underlying scientific basis of the novel evaluation method. The second manipulation—evaluator credentials—was used to measure the extent to which perceptions of the evaluator and their opinion were influenced by the evaluator’s credibility. In both conditions, the evaluator had a doctoral degree and specialized training in forensic psychology; in the low credibility condition, the evaluator had one year of evaluator experience. In the high credibility condition, the evaluator had “over 15 years” of experience. Expert witnesses’ credentials have influenced perceptions of credibility and trustworthiness in previous studies (Flick et al., 2022), and in Study One, low source quality resulted in a stronger CK effect. We would therefore expect it to also influence perceptions of evaluators in a legal context. Lastly, the evaluator’s final opinion of whether the individual acted intentionally or not was included to assess the extent to which the CK effect changes participants’ agreement with the expert’s opinion. This will help discern whether the CK effect is not only present, but also results in differential outcomes in legal cases. Gowensmith et al. (2012) found evidence that in the majority of cases, judges agree with evaluator opinions; therefore, we expect a similar pattern to emerge in Study Two.

Participants who previously indicated within the Prolific system that they were 18 years of age or older, spoke English, and lived in the United States received an email with a brief statement outlining the project topic and aims, with an invitation to participate. Interested participants were directed to an online survey created using Qualtrics online survey platform, where they reviewed the informed consent detailing voluntary participation and minimal anticipated risks as a result of their participation. Participants who consented were then randomly assigned to one of eight conditions where they read a mock court hearing vignette. After reading the vignette, participants answered questions assessing the CK effect, perceptions of the evaluator and their testimony, and opinions regarding the defendant’s criminal responsibility and guilt. Finally, participants answered demographic questions. Participants’ responses were screened to ensure eligibility criteria were met and attention checks were passed. Following confirmation of these criteria, participants received a total of $1.81 for their time.

##### Study Two Witness Credibility Scale

The Witness Credibility Scale (WCS; Brodsky et al., 2010) is a 20-item scale used in this study to measure perceptions of the expert’s overall credibility. The WCS produces a total score and subscale scores including Confident (e.g., “self-assured”), Likable (e.g., “pleasant”), Trustworthy (e.g., “honest”), and Knowledgeable (e.g., “wise”); however, the current study only utilized the last three factors, as they were the most applicable to written vignettes without visual stimuli. Items on the WCS reference participants’ opinions on the extent to which the expert possesses various qualities known to increase credibility; each item is rated on a 10-point Likert scale from least (1) to most (10). Items within each factor are summed to create their respective subscale score. Subscale scores range from 5 to 50, with higher scores reflecting an increased possession of that quality.

##### Study Two Perceived Homophily Measure

The Perceived Homophily Measure (PHM; McCroskey et al., 1975) was used in this study to assess participants’ perceptions of the expert being a member of their own social community. The PHM scale asks participants to rate their perceived similarity to another person (in this case, the expert witness) in various aspects on a seven-point Likert scale, ranging from least similar (1) to most similar (7). The PHM has four distinct factors: Attitude, Background, Value, and Appearance; however, the current study only utilized the first two factors, as they were the most applicable to written vignettes without visual stimuli. The Attitude subscale has four items, including “Doesn’t think like me” (1) to “Thinks like me” (7). Similarly, the Background subscale contains four items, such as “Economic situation different from mine (1) to “Economic situation like mine” (7). Items within each factor are summed to create their respective subscale score, with higher values indicating greater perceived homophily, or sameness, to the individual referenced.

##### Study Two Demographic Questionnaire

Participants were asked questions about their age, race, gender, and other demographic characteristics. Participants were also asked about their educational, professional, and legal history. For all demographic questions, participants were given the option of “prefer not to respond”.

##### Study Two Researcher-Derived Questions

In line with Study One, questions were included to measure participants’ beliefs and trust in the fake scientific information, as well as their perceived ability to understand and explain the fake scientific information. These questions were presented on a five-point Likert-type scale from 1 (not well at all) to 5 (extremely well). Similarly, participants were asked “Did you receive an explanation on how Cognitive Intent Imaging (CII) works” on a scale from 1 (no explanation at all) to 5 (a complete, thorough explanation).

Regarding participants’ legal opinions, they were asked “Do you agree with the expert witness’s opinion about Mr. Smith’s ability to act intentionally at the time of the offense?” and “Do you think that Mr. Smith should be found guilty of the voluntary manslaughter of his neighbor?” They were also asked to assign weight to the expert’s testimony, the fake scientific information, and the expert’s experience on a sliding scale from 0 (no weight at all) to 100 (all of the weight any evidence in the case could receive).

One attention check (i.e., “Please select ‘No’ for this question”) and two manipulation checks (i.e., “What was the expert witness’s opinion about Mr. Smith’s ability to act intentionally?” and “How many years of experience did the expert witness have?”) were included to ensure high-quality data were collected.

#### 4.2.2. Study Two Results

All dependent measures met the general assumptions for ANOVA testing. Study Two did not have any within-subjects components and, therefore, collapsing data was unnecessary. For this reason, mean and standard deviation values in Study Two represent true participant responses, rather than changes in participant responses between conditions. With the exception of the Background subscale of the Perceived Homophily Measure, all dependent variables were positively correlated with each other, with *p* values ranging from <0.001 to 0.03. Descriptive statistics for all study measures are provided in Table 4.

##### Study Two Expert Understanding

A multivariate analysis of variance (MANOVA) was used to test the relationship between evaluator understanding and participants’ perceived ability to understand and explain the scientific information, as well as participants’ belief in and trust in the scientific information. Results demonstrated an overall significant difference in perceptions of scientific information and perceptions of the evaluator based on evaluator understanding, Pillai’s Trace = 0.17, *F*(11, 382) = 7.03, *p* < 0.001, η^2^ = 0.17. Specifically, participants rated their perceived understanding of the scientific information significantly higher when they were told the evaluator could understand and explain the scientific information (*M* = 2.07, *SE* = 0.07) compared to when the evaluator could not, (*M* = 1.52, *SE* = 0.07), *F*(1, 396) = 30.54, *p* < 0.001, η^2^ = 0.07. Participants also had greater trust in the scientific information when they were told the evaluator could understand and explain it, (*M* = 2.05, *SE* = 0.07) compared to when the evaluator could not, (*M* = 1.55, *SE* = 0.08), *F*(1, 396) = 22.89, *p* < 0.001, η^2^ = 0.06. Their belief that the scientific information was real was significantly stronger when the evaluator could understand and explain the scientific information (*M* = 2.17, *SE* = 0.08), compared to when the evaluator could not (*M* = 1.81, *SE* = 0.09), *F*(1, 396) = 9.69, *p* < 0.01, η^2^ = 0.02. Finally, participants’ perceived ability to explain the scientific information was rated significantly higher when they were told the evaluator could understand and explain the scientific information (*M* = 1.88, *SE* = 0.07), compared to when the evaluator could not (*M* = 1.50, *SE* = 0.07), *F*(1, 396) = 15.44, *p* < 0.001, η^2^ = 0.04. Put together, these findings suggest a successful replication of Study One within the legal context.

Concerning participants’ perceptions of the evaluator, when participants were told the evaluator could understand and explain the scientific information, they rated the evaluator higher in areas of Likeability (*M* = 30.75, *SE* = 0.58 vs. *M* = 28.84, *SE* = 0.63), *F*(1, 396) = 4.93, *p* = 0.03, η^2^ = 0.01, Trust (*M* = 30.65, *SE* = 0.73 vs. *M* = 23.62, *SE* = 0.80), *F*(1, 396) = 42.16, *p* < 0.001, η^2^ = 0.10, and knowledge subscales (*M* = 32.92, *SE* = 0.73 vs. *M* = 26.72, *SE* = 0.80), *F*(1, 396) = 32.83, *p* < 0.001, η^2^ = 0.08, compared to when the evaluator could not understand or explain the scientific information. Participant scores on the PHM Attitude subscale were significantly higher when they were told the evaluator could understand and explain the scientific information (*M* = 15.35, *SE* = 0.29) compared to when the evaluator could not (*M* = 13.48, *SE* = 0.31), *F*(1, 396) = 19.75, *p* < 0.001, η^2^ = 0.05, suggesting participants felt more socially distant from the evaluator when they could not understand or explain the scientific information. There was no difference in PHM Background subscale scores depending on the evaluator’s ability to understand and explain the scientific information, *F*(1, 396) = <0.01, *p* = 0.96, η^2^ < 0.01.

##### Study Two Expert Credentials

The MANOVA between evaluator credentials and participant perceptions of the scientific information and expert witness was not significant, *Pillai’s Trace* = 0.03, *F*(9, 386) = 1.20, *p* = 0.30, η^2^ = 0.03. For this reason, no individual tests within the MANOVA were interpreted. Further, there was not a significant interaction effect between evaluator understanding and evaluator credentials on perceived understanding, *Pillai’s Trace* = 0.01, *F*(11, 382) = 0.49, *p* = 0.91, η^2^ = 0.01.

##### Study Two Weight of Expert Testimony and Scientific Information

Participants assigned more weight to the expert testimony when they were told the evaluator could understand and explain the scientific information (*M* = 47.19, *SE* = 1.80), compared to when the evaluator could not (*M* = 32.23, *SE* = 1.95), *F*(1, 396) = 31.61, *p* < 0.001, η^2^ = 0.08. Likewise, weight assigned to the scientific information itself was higher when participants were told the evaluator could understand and explain it (*M* = 40.11, *SE* = 1.95), than when the evaluator could not (*M* = 23.00, *SE* = 2.11), *F*(1, 396) = 35.53, *p* < 0.001, η^2^ = 0.08.

##### Study Two Mediating Role of Perceived Social Distance

Structural Equation Modeling (SEM) mediation models were used to test conceptual relationships between expert understanding, perceived social distance from the expert, and participant understanding, ability to explain, belief in, and trust in the scientific information. The direct effect of expert understanding on perceived understanding (*b* = 0.46, *SE* = 0.10, *t* = 4.52, *p* < 0.001), trust (*b* = 0.37, *SE* = 0.10, *t* = 3.69, *p* < 0.05), believability (*b* = 0.27, *SE* = 0.11, *t* = 2.34, *p* < 0.001), and ability to explain (*b* = 0.29, *SE* = 0.10, *t* = 2.95, *p* < 0.01) were all significant. Similarly, the indirect effects of the Attitude scale of the PHM were significant, indicating that perceived social distance in terms of the evaluator’s attitude mediated relationships between evaluator understanding and perceived understanding (95% CI [0.04, 0.16]), trust (95% CI [0.07, 0.22]), believability (95% CI [0.04, 0.19]), and ability to explain (95% CI [0.04, 0.17]). The Background scale of the PHM did not significantly mediate these relationships. See Table 5 for model details.

##### Study Two Mediating Role of Perceived Credibility

SEM mediation models were also used to evaluate whether the relationship between evaluator understanding and perceptions of scientific information varied by ratings of witness credibility. The direct effect of evaluator understanding on participant understanding (*b* = 0.35, *SE* = 0.11, *t* = 3.30, *p* < 0.001) and trust (*b* = 0.21, *SE* = 0.10, *t* = 2.04, *p* < 0.05) were significant; however, the direct effect of evaluator understanding on perceived believability (*b* = 0.09, *SE* = 0.12, *t* = 0.79, *p* = 0.43), and ability to explain (*b* = 0.19, *SE* = 0.10, *t* = 1.85, *p* = 0.06) were not. Ratings on the trustworthiness subscale of the WCS mediated the relationships between evaluator understanding and participant understanding (95% CI [0.01, 0.34]), trust (95% CI [0.14, 0.50]), believability (95% CI [0.12, 0.55]), and ability to explain (95% CI [0.06, 0.37]). The mediating effect of the likability subscale of the WCS was marginal in that the 95% confidence interval did not contain zero, but the *p*-value did not indicate statistical significance. Ratings on the knowledge subscale of the WCS did not significantly mediate these relationships. See Table 6 for model details.

##### Study Two Perceptions of Expert Opinion

Roughly half (52%) of participants indicated they believed the defendant should be found guilty of voluntary manslaughter. A chi-square test evaluated whether participants would more often think the defendant should be found guilty when the judge ruled the defendant criminally responsible. The test showed that the number of participants who thought the defendant should be guilty did not differ by the judge’s opinion of criminal responsibility, X(1, N = 396) = 3.79, *p* = 0.06.

The majority of participants (60.9%) disagreed with the evaluator’s opinion about criminal responsibility. A chi-square test assessed the influence of expert understanding of the scientific information on participants’ opinions regarding criminal responsibility. Results indicated participants agreed with the evaluator’s opinion regarding criminal responsibility more often when the evaluator reportedly understood how the scientific information worked, *X*(1, N = 396) = 11.25, *p* < 0.001.

#### 4.2.3. Study Two Discussion

Study Two aimed to replicate Study One in a legal context and to extend the CK effect to understand how it ultimately impacts decision-making in legal settings. Hypothesis 1 was supported in that when the forensic evaluator reported understanding the scientific information and being able to explain how it worked, participants’ perceived understanding, ability to explain, trust in the information, and belief that the science was real all increased. We also found that participants’ ratings of the evaluator’s credibility increased, they assigned more weight to the evaluator’s testimony in making final decisions about the defendant, and they agreed more often with the evaluator’s opinion regarding the defendant’s criminal responsibility.

The CK effect in Study Two stands in stark contrast to other studies measuring evaluator credibility and scientific testimony. In one study using real case details and scientific testimony without CK effect manipulations, experts with higher degrees and high-quality testimony were perceived as more credible than those with lower degrees or with lower-quality testimony (Flick et al., 2022). Another study by Koehler et al. (2016) found that jurors weighed scientific information more heavily when presented by an expert with more experience. Therefore, the CK effect seems to reverse the traditional relationship between scientific information, source credibility, and perceptions of credibility and weight given during decision-making; when the CK effect is present, the actual credibility of the source matters less than the evaluator’s self-reported understanding of scientific information. If these findings are representative of what occurs in legal cases, then expert witnesses with self-reported confidence and comfort level with scientific information of unknown quality could sway juror decisions. Additional research in new samples confirming these findings and examining their impact on more detailed aspects of witness credibility, such as perceptions of warmth and competence, will elucidate this further.

Study Two findings additionally offer insight into the nuances of the CK effect and underlying theory. Mediation models showed that the relationship between evaluator understanding and participant understanding was explained by the extent to which participants perceived themselves to be similar in attitude to the evaluator. This provides evidence of construct validity for the CK effect, as the perception of shared community drove increases in participant understanding. Interestingly, although perceptions of the trust in the evaluator (via the WCS Trust subscale) also explained participant understanding, source quality was unrelated to dependent variables (including ratings of evaluator credibility and perceived similarity to the evaluator, which drive ratings of participant understanding). This suggests that perceptions of sameness to the information source, rather than true source credibility, motivate the CK effect. Given that this was also a hypothesized explanation for findings in Study One, there is increasing evidence that perceptions of shared community are more powerful than objective source credibility when deciding to trust and believe novel scientific information.

There was also evidence in Study Two that the CK effect may represent a reciprocal, rather than a strictly linear, process. For example, perceptions of witness credibility—including likability, trust, and knowledge—as well as perceived social distance from the evaluator, changed as a result of evaluator understanding. If the relationship between expert understanding and perceptions of community were linear, then these would not change as a result of expert understanding. More research on the nature and time order of these relationships is needed to better understand what the true function of these relationships is. Another consideration for the CK effect and theory is that Study Two examined the CK effect in reference to a single scientific expert’s perceived understanding, rather than “scientists” generally in Study One. Both studies found evidence for the CK effect in that the scientist(s)’ understanding increased participant understanding, trust, and believability; however, it is possible that the CK effect may operate differently when referencing a single scientist versus an entire scientific community.

These results beg the question of how researchers can help judges and attorneys protect courtrooms from being subjected to biased scientific information and insufficiently supported testimony. It is particularly concerning that participants agreed with the expert more often when the expert reportedly understood how the scientific information worked. Research shows judges oftentimes agree with forensic evaluators’ opinions (97.7% when evaluator opinions are unanimous; Gowensmith et al., 2012), but if the CK effect changes how often jurors agree with the evaluator’s opinion, it may also change the frequency with which judges agree. In a time post-Daubert, when judges are called to be the “gatekeepers” of the courtroom, if they are just as susceptible to the CK effect, then all legal fact finders may be at risk of biased decision-making as a result of faulty scientific information and testimony.

Contrary to Study One and Hypothesis 2, there was no significant influence of expert credentials on perceptions of the scientific information, nor was there an interaction between expert understanding and expert credentials. In other words, the CK effect was not any stronger when the expert was less experienced. This could be explained by the fact that both conditions depicted a doctoral-level expert whom participants perceived as credible, even though one expert had fewer years of experience. It is possible that the difference in source credibility was marginal in this scenario. The lack of effect could also be explained by the fact that the source quality manipulation was not strong enough; indeed, there were 32 participants who failed both manipulation checks, and several more who failed at least one. Thus, the CK effect may be stronger when the source quality is low, but a replication study with a stronger manipulation of source quality is needed to confirm such a finding.

Our hypothesis that perceived social distance would be lower, and credibility higher, when the evaluator was less experienced (H3) was not supported in Study Two. Similarly to Study One, there was no main effect of expert credibility on perceptions of either the scientific information shared or the expert witness. This finding was unexpected, as CK theory suggests that it is dependent upon perceptions of the expert, which moderated the CK effect in Study One. However, given that perceived social distance mediated the relationship between expert understanding and perceptions of the scientific information, it is likely true that the relationship between source credibility and the CK effect is less about the source’s objective credibility and more about perceptions of being similar to one another, or within the same “community”. Source credibility and perceived similarity were not related constructs in Study Two.

Lastly, we expected that participants’ perceived similarity to the expert would mediate the relationship between expert understanding and perceptions of the scientific information (H4). In support of this, we found that participants’ perceived similarity to the expert in terms of attitude or thinking style mediated the relationship between expert understanding and participant understanding of, believability of, trust in, and ability to explain the scientific information. This provides empirical evidence for the theoretical underpinnings of the CK effect, which are that individuals accept another person’s self-reported knowledge of scientific information because the individual perceives them to be within the same “community” of shared knowledge. While Study Two still needs to be replicated, it offers useful information to begin designing mitigation techniques that can be used in legal settings. It is possible that attorney questioning or jury instructions that reduce the impression the expert is in the same “community” or has different, specialized knowledge that jurors do not have may help to reduce the impact the CK effect has on decision-making.

Although this study builds upon Study One and provides important findings to further research on expert witness testimony, the use of scientific information in court, and the CK effect broadly, some limitations reduce the ability to generalize its findings. One limitation is that of reduced ecological validity—there were some details included in the vignette necessary for eliciting measurable responses that may not be included in a typical expert testimony or trial scenario. Or, they would not be present in the same way they appeared in Study Two. For example, evaluators do not typically offer an ultimate opinion on a defendant’s mental state during testimony, and it is unlikely an expert would testify they “did not understand” the science behind a technique they used. Further, it is unclear if judges may secondarily represent an expert to jurors—because they too have expertise, and could fall within the same community as jurors, but are relying upon other scientists for their own understanding of scientific information. Ultimately, jurors will come across the same information provided in the vignette through cross-examination, testimony from opposing experts whose opinions or techniques differ, and when judges offer their ruling on matters involving mental state examinations. Future studies should nonetheless aim for more realistic experimental materials, such as videos or court transcripts, and testing of whether information influences jurors further after judge’s decisions are made, to determine if the CK effect is also elicited in more realistic contexts.

Another limitation is that this study focused on a forensic evaluator as the expert and did not consider other types of scientific information or experts. It also did not compare perceptions of the fake scientific information—“Cognitive Intent Imaging”— to real techniques employed by forensic evaluators to determine if the CK effect is also present with real, legitimized scientific techniques. Research is therefore needed to find evidence of the CK effect in other samples using other types of forensic experts and scientific information, both real and fake. Similarly, research on methods of maximizing jurors’ accurate use of forensic science testimony in the face of the CK effect is needed; existing literature on maximizing juror comprehension of information may offer a starting point for such research (see Eldridge, 2019 for a review). An ecologically valid study that employs videos of a trial could be used to elicit the CK effect, then control conditions with no mitigation strategies can be compared to other conditions containing different mitigation strategies.

Another limitation of Study Two is that similar to Study One, we did not test the extent to which the CK effect is the product of impression management by participants. However, in Study Two we found the CK effect was partly explained by perceptions of social distance from the evaluator. If the CK effect was primarily due to impression management, then it would theoretically be unrelated to perceptions of social distance because these constructs are separate. Nevertheless, research is needed to continue testing the CK effect and to rule out alternative explanations for its presence in both studies. A replication of Study One or Two with inclusion of questions around positive impression management or deception could easily allow researchers to address this limitation.

## 5. Conclusions

Studies One and Two successfully replicated those conducted by Sloman and Rabb (2016), taking important next steps in the exploration of the CK effect. We found that not only did the CK effect persist in new samples and with new experimental materials, but in Study Two it was explained in part by perceptions of social distance from the expert, which has important implications for the conceptualization of the CK effect and what motivates observed increases in perceptions of understanding, trust, believability, and ability to explain. These findings also opened the door to new research in expert testimony and scientific information in courts by finding evidence for the CK effect in the context of a mock criminal responsibility hearing with a forensic evaluator as an expert witness. They have important implications for courtroom personnel because juries and judges rely upon experts to make use of scientific processes and information. These studies demonstrate the fallible nature of this process by showing that juries and judges need not receive an explanation from an expert witness as to how the scientific processes work before they offer trust and give more weight to the scientific information. In a generation where science and technology change rapidly—and now includes concepts like machine learning, augmented reality, and artificial intelligence—the field of jury decision-making and use of scientific information is similarly changing. As long as jury decision-making science lags behind these novel developments and techniques, courtrooms will remain underserved by researchers.

Certain mitigating strategies may help to insulate jurors and judges from blindly trusting faulty scientific information and/or testimony in the context of the CK effect. First, attorneys and judges may pay close attention to expert testimony for statements that could invite the CK effect, such as vague reports of “understanding” how particular scientific techniques work without explaining such details on the stand. Attorneys may make Daubert challenges more often in court rather than relying upon generalized statements from the expert that their techniques meet admissibility thresholds. Lastly, forensic evaluators, when testifying, may take it upon themselves to provide more details regarding their evaluation procedures and the scientific support for their techniques to mitigate the CK effect and assist fact finders in relying upon real scientific justification. Ultimately, although additional research is sorely needed in this emerging area of bias research, these studies offer much in helping researchers and experts understand the relevance of the CK effect to courtroom settings.

## Figures and Tables

**Table 1:** Demographic characteristics of Study One sample (N = 291).

Demographic	N	%	Demographic	N	%
			**Race/Ethnicity**		
**Sex**			African American/Black	24	8.2
Male	161	55.3	Middle Eastern/North African	1	<1
Female	130	44.7	Asian/Pacific Islander	28	9.6
			White/Caucasian	208	71.5
**Political Views**			Hispanic/Latino/Central/South American	21	7.2
Very Conservative	17	5.8	Other	9	3.1
Somewhat Conservative	35	12.0			
Moderate, Leaning Conservative	18	6.2	**Education**		
Moderate	65	22.3	High school graduate	49	16.8
Moderate, Leaning Liberal	37	12.7	Some college	80	27.5
Somewhat Liberal	60	20.6	Associate’s degree	32	11.0
Very Liberal	59	20.3	Bachelor’s degree	106	36.4
			Master’s degree	20	6.9
			Doctoral degree	4	1.4

**Table 2:** Descriptive statistics of dependent measures.

DV	*M*	*SD*
**Rock Scenario**		
Perceived understanding	1.62	1.01
Perceived ability to explain	1.62	1.07
Believability	2.30	1.17
Trustworthiness	2.61	1.20
Received explanation	0.49	0.50
**Stalactites Scenario**		
Perceived understanding	1.57	0.96
Perceived ability to explain	1.48	0.92
Believability	2.83	1.06
Trustworthiness	3.01	1.02
Received Explanation	0.50	0.50

Note. Measures ranged from 1–5 on a Likert scale.

**Table 3:** Demographic characteristics of Study Two sample (N = 396).

Demographic	N	%	Demographic	N	%
**Gender**			Ethnicity		
Male	162	40.9	Hispanic/Latine/Spanish	43	10.9
Female	222	56.1	Not applicable	352	88.9
Non-binary	8	2.0	Prefer not to respond	1	<1
Gender Fluid	1	<1			
Transgender Nonbinary	1	<1	**Education**		
Prefer not to respond	2	<1	Less than high school	1	<1
			diploma		
**Race**			High school	44	11.1
African American/Black	67	16.9	degree/equivalent		
American Arab/Middle	2	<1	Some college, no degree	82	20.7
Eastern/North African			Technical certificate or	8	2.0
Asian/Asian American	12	3.0	training		
Native American/Alaskan	4	1.0	Associate’s degree	39	9.8
Native			Bachelor’s degree	144	36.4
White	277	69.9	Master’s degree	58	14.6
Multiracial	22	5.6	Professional degree	4	1.0
Other	6	1.5	Doctorate	14	3.5
Prefer not to respond	6	1.5	Other	1	<1
			Prefer not to respond	1	<1
					
			**Political affiliation**		
			Democratic	177	44.7
			Republican	101	25.5
			Independent	106	26.8
			Prefer not to respond	12	3.0

**Table 4:** Correlational relationships between dependent variables in Study Two.

Scale	1	2	3	4	5	6	7	8	9	10	11	12	13	14
1. Understanding	-	-	-	-	-	-	-	-	-	-	-	-	-	-
2. Ability to Explain	0.86 ***	-	-	-	-	-	-	-	-	-	-	-	-	-
3. Trust	0.68 ***	0.71 ***	-	-	-	-	-	-	-	-	-	-	-	-
4. Believability	0.56 ***	0.56 ***	0.72 ***	-	-	-	-	-	-	-	-	-	-	-
5. Others’ Understanding	0.68 ***	0.67 ***	0.67 ***	0.58 ***	-	-	-	-	-	-	-	-	-	-
6. Explanation	0.56 ***	0.55 ***	0.49 ***	0.39 ***	0.45 ***	-	-	-	-	-	-	-	-	-
7. WCS Likability	0.12 *	0.11 *	0.14 **	0.14 **	0.15 **	0.12 *	-	-	-	-	-	-	-	-
8. WCS Trust	0.32 ***	0.30 ***	0.40 ***	0.34 ***	0.35 ***	0.31 ***	0.67 ***	-	-	-	-	-	-	-
9. WCS Knowledge	0.31 ***	0.27 ***	0.37 ***	0.30 ***	0.32 ***	0.28 ***	0.64 ***	0.90 ***	-	-	-	-	-	-
10. PHM Attitude	0.29 ***	0.29 ***	0.36 ***	0.25 ***	0.30 ***	0.28 ***	0.29 ***	0.14 **	0.46 ***	-	-	-	-	-
11. PHM Background	0.18 ***	0.20 ***	0.23 ***	0.14 **	0.19 ***	0.14 **	0.03	0.14 **	0.13 *	0.39 ***	-	-	-	-
12. Testimony Weight	0.47 ***	0.43 ***	0.58 ***	0.45 ***	0.45 ***	0.40 ***	0.31 ***	0.64 ***	0.60 ***	0.39 ***	0.19 ***	-	-	-
13. Science Weight	0.52 ***	0.52 ***	0.70 ***	0.59 ***	0.54 ***	0.47 ***	0.22 ***	0.54 ***	0.48 ***	0.43 ***	0.21 ***	0.77 ***	-	-
14. Experience Weight	0.30 ***	0.28 ***	0.39 ***	0.32 ***	0.31 ***	0.26 ***	0.35 ***	0.58 ***	0.59 ***	0.32 ***	0.10	0.68 ***	0.56 ***	-
*M*	1.82	1.72	1.84	2.02	2.07	2.18	29.94	27.50	30.18	14.49	13.26	40.66	32.48	50.54
*SD*	1.01	0.97	1.06	1.15	1.05	0.96	8.51	11.23	11.10	4.24	4.38	27.28	29.45	26.87

Note. WCS = Witness Credibility Scale; PHM = Perceived Homophily Measure; * *p* < 0.05; ** *p* < 0.01; *** *p* < 0.001.

**Table 5:** SEM model with PHM subscales in Study Two.

DV	Path	b	SE	*p*	95% CI	
					Lower	Upper
Perceived Understanding (PU)	Direct Effect	0.228	0.051	<0.001 *	0.125	0.324
	EU → PHM A → PU	0.044	0.015	0.003 *	0.019	0.078
	EU → PHM B → PU	0.001	0.006	0.927	−0.011	0.014
	Total Indirect Effect	0.045	0.017	0.008 *	0.013	0.080
	Total Effect	0.273	0.049	<0.001 *	0.173	0.365
Trust	Direct Effect	0.178	0.048	<0.001 *	0.081	0.272
	EU → PHM A → Trust	0.062	0.017	<0.001 *	0.032	0.100
	EU → PHM B → Trust	0.001	0.007	0.925	−0.013	0.015
	Total Indirect Effect	0.063	0.020	0.002 *	0.026	0.104
	Total Effect	0.241	0.048	<0.001 *	0.147	0.337
Believability (Bel.)	Direct Effect	0.116	0.049	0.019 *	0.018	0.212
	EU → PHM A → Bel.	0.044	0.016	0.006 *	0.018	0.082
	EU → PHM B → Bel.	0.001	0.004	0.939	−0.007	0.011
	Total Indirect Effect	0.045	0.017	0.009 *	0.015	0.082
	Total Effect	0.160	0.049	0.001 *	0.064	0.256
Ability to Explain (Ab. To Exp.)	Direct Effect	0.149	0.051	0.003 *	0.048	0.246
	EU → PHM A → Ab. to Exp.	0.048	0.016	0.002 *	0.022	0.085
	EU → PHM B → Ab. to Exp.	0.001	0.007	0.926	−0.012	0.015
	Total Indirect Effect	0.049	0.018	0.008 *	0.015	0.087
	Total Effect	0.198	0.049	<0.001 *	0.099	0.291

Note. EU = expert understanding; PHM A = Perceived Homophily Measure—Attitude subscale; PHM B = Perceived Homophily Measure—Background subscale; * statistically significant.

**Table 6:** SEM model with WCS subscales in Study Two.

DV	Path	b	SE	*p*	95% CI	
					Lower	Upper
Perceived Understanding (PU)	Direct Effect	0.170	0.052	0.001 *	0.070	0.273
	EU → WCS L → PU	−0.017	0.011	0.113	−0.048	−0.003
	EU → WCS T → PU	0.082	0.037	0.029 *	0.008	0.156
	EU → WCS K → PU	0.034	0.031	0.282	−0.022	0.100
	Total Indirect Effect	0.098	0.019	<0.001 *	0.066	0.140
	Total Effect	0.268	0.049	<0.001 *	0.172	0.361
Trust	Direct Effect	0.095	0.047	0.044 *	0.006	0.193
	EU → WCS L → Trust	−0.024	0.013	0.054	−0.055	−0.005
	EU → WCS T → Trust	0.137	0.037	<0.001 *	0.065	0.210
	EU → WCS K → Trust	0.021	0.029	0.484	−0.030	0.086
	Total Indirect Effect	0.133	0.023	<0.001 *	0.093	0.182
	Total Effect	0.228	0.047	<0.001 *	0.140	0.323
Believability (Bel.)	Direct Effect	0.039	0.049	0.430	−0.057	0.136
	EU → WCS L → Bel.	−0.017	0.010	0.099	−0.046	−0.003
	EU → WCS T → Bel.	0.131	0.040	0.001	0.055	0.211
	EU → WCS K → Bel.	0.002	0.030	0.960	−0.054	0.066
	Total Indirect Effect	0.115	0.023	<0.001 *	0.075	0.166
	Total Effect	0.154	0.047	0.001 *	0.062	0.246
Ability to Explain (Ab. to Exp.)	Direct Effect	0.094	0.051	0.066	−0.005	0.196
	EU → WCS L → Ab. to Exp.	−0.017	0.011	0.109	−0.048	−0.003
	EU → WCS T → Ab. to Exp.	0.101	0.036	0.005 *	0.033	0.176
	EU → WCS K → Ab. to Exp.	0.014	0.031	0.646	−0.044	0.079
	Total Indirect Effect	0.098	0.018	<0.001 *	0.067	0.138
	Total Effect	0.192	0.049	<0.001 *	0.099	0.288

N Note. EU = expert understanding; WCS L = Witness Credibility Scale—Likability subscale; WCS T = Witness Credibility Scale—Trust subscale; WCS K = Witness Credibility Scale—knowledge subscale; * statistically significant.

## Data Availability

Some original data and supporting materials for this study are openly available on Open Science Framework (OSF) at https://doi.org/10.17605/OSF.IO/83GSM (accessed on 1 August 2025).
